# Observation of Bacterial Type I Pili Extension and Contraction under Fluid Flow

**DOI:** 10.1371/journal.pone.0065563

**Published:** 2013-06-14

**Authors:** Dilia E. Rangel, Nathaly Marín-Medina, Jaime E. Castro, Andrés González-Mancera, Manu Forero-Shelton

**Affiliations:** 1 Department of Mechanical Engineering, Universidad de los Andes, Bogotá, Colombia; 2 Department of Physics, Universidad de los Andes, Bogotá, Colombia; Charité, Campus Benjamin Franklin, Germany

## Abstract

Type I pili are proteinaceous tethers that mediate bacterial adhesion of uropathogenic *Escherichia coli* to surfaces and are thought to help bacteria resist drag forces imparted by fluid flow via uncoiling of their quaternary structure. Uncoiling and recoiling have been observed in force spectroscopy experiments, but it is not clear if and how this process occurs under fluid flow. Here we developed an assay to study the mechanical properties of pili in a parallel plate flow chamber. We show that pili extend when attached *E. coli* bacteria are exposed to increasing shear stresses, that pili can help bacteria move against moderate fluid flows, and characterize two dynamic regimes of this displacement. The first regime is consistent with entropic contraction as modeled by a freely jointed chain, and the second with coiling of the quaternary structure of pili. These results confirm that coiling and uncoiling happen under flow but the observed dynamics are different from those reported previously. Using these results and those from previous studies, we review the mechanical properties of pili in the context of other elastic proteins such as the byssal threads of mussels. It has been proposed that the high extensibility of pili may help recruit more pili into tension and lower the force acting on each one by damping changes in force due to fluid flow. Our analysis of the mechanical properties suggests additional functions of pili; in particular, their extensibility may reduce tension by aligning pili with the direction of flow, and the uncoiled state of pili may complement uncoiling in regulating the force of the terminal adhesin.

## Introduction

Bacterial adhesion assures attachment to host cell surfaces and is the first step of the infection process. When bacteria are immersed in a moving fluid, this adhesion process takes place under shear stress and the resulting drag forces when bacteria attach to the surface. Some bacteria have developed organelles which help them withstand these forces such as P and type I pili in uropathogenic *Escherichia coli* [1], CFA/I fimbria in enterotoxigenic *Escherichia coli* [2], type I and type IV pili in *Xylella fastidiosa* [3] and PI-2A pilus in *Streptococcus agalactiae* [4]. In uropathogenic *E. coli*, P and type I pili help bacteria bind and invade uroepithelial cells in the adverse conditions of the urinary tract [5]–[7]. The mechanical properties of these two types of pili have been studied by means of optical tweezers (OT) [8]–[14] and atomic force microscopy (AFM) [15], [16] but it is not clear how these properties translate into an environment closer to physiological settings where forces may be applied differently.

Type I pili are surface filamentous organelles anchored to the outer membrane of *E. coli*, ∼1 µm long and 7 nm in diameter in their coiled state. The bulk of these pili is composed of FimA monomers which are arranged in a tight right-handed helical rod [17], [18], strongly bound to each other by donor-strand exchange [19], [20]. Their apex is composed of the subunits FimF, FimG [21] and the FimH adhesin at the tip [22] which shows affinity for mannosylated proteins [23] like those found on the surface of uroepithelial cells. Previous studies have shown that type I pili present a mechanism to optimize the adhesion process under flow conditions: the bond between their adhesin and the receptor on the host cell behaves as a catch-bond, meaning that its lifetime increases with increasing force up to a maximum lifetime at an optimal force, and then decreases [24]–[26].

Another mechanism that is expected to improve the adhesive properties of bacteria is the extension of pili under force. AFM [15] and OT [11]–[13], [27] experiments have shown that type I pili elongate significantly at forces above ∼50 pN and contract at forces below ∼25 pN due to uncoiling and coiling of their quaternary structure respectively. Simulations have shown that this mechanism can reproduce enhanced adhesion [28], [29]. Simulations also suggest that they can work as dampers for sudden changes in flow [30]. However, it is not clear so far how the mechanics of type I pili operate and cooperate when mediating bacterial adhesion under flow as opposed to the controlled settings of OT and AFM. OT experiments are performed near equilibrium and AFM experiments have been done at moderate uncoiling rates, whereas in the flow conditions being studied high uncoiling rates can be achieved. It is also not possible with either experimental technique to mimic the torques and other possible effects, such as changes in geometry and pili buckling that may arise from a bacterium moving in fluid flow.

Although much has been said about the role of uncoiling and the mechanics of the adhesive tip, little has been said about the function of the uncoiled state of pili. Our experiments model flow conditions in a parallel flow chamber by generating shear stresses of similar magnitude to those expected physiologically [31] to study the response of *E. coli* attached to surfaces via type I pili to changes in shear stress. In order to focus on the mechanical properties of type I pili, we developed an assay that sustains long-lived FimH-receptor adhesions. By tracking bacteria we obtain curves of their position along the axis of fluid flow as a function of time. Bacterial displacements due to pili extension or contraction after changes in flow rates are compared with previous AFM and OT data, and discussed. These results hint to the relevance of the uncoiled state in helping damp changes in fluid flow. Finally, by examining the mechanical properties of both coiled and uncoiled pili in the context of the byssal filaments of mussels, we are able to present a consistent picture from which we propose new functionalities for the uncoiled state of pili, as well as for coiling and uncoiling in the context of the bacterial attachment process.

## Results

### Bacterial Displacement in the Direction of Flow is Consistent with the Uncoiling of Pili

In order to ensure that bacterial movement observed in the flow chamber videos is caused by bacterial displacement, we first performed a control that ensured that chamber movement during flow changes did not result in the apparent movement of bacteria (see supplementary Text S1 for details).

**Figure 1:**
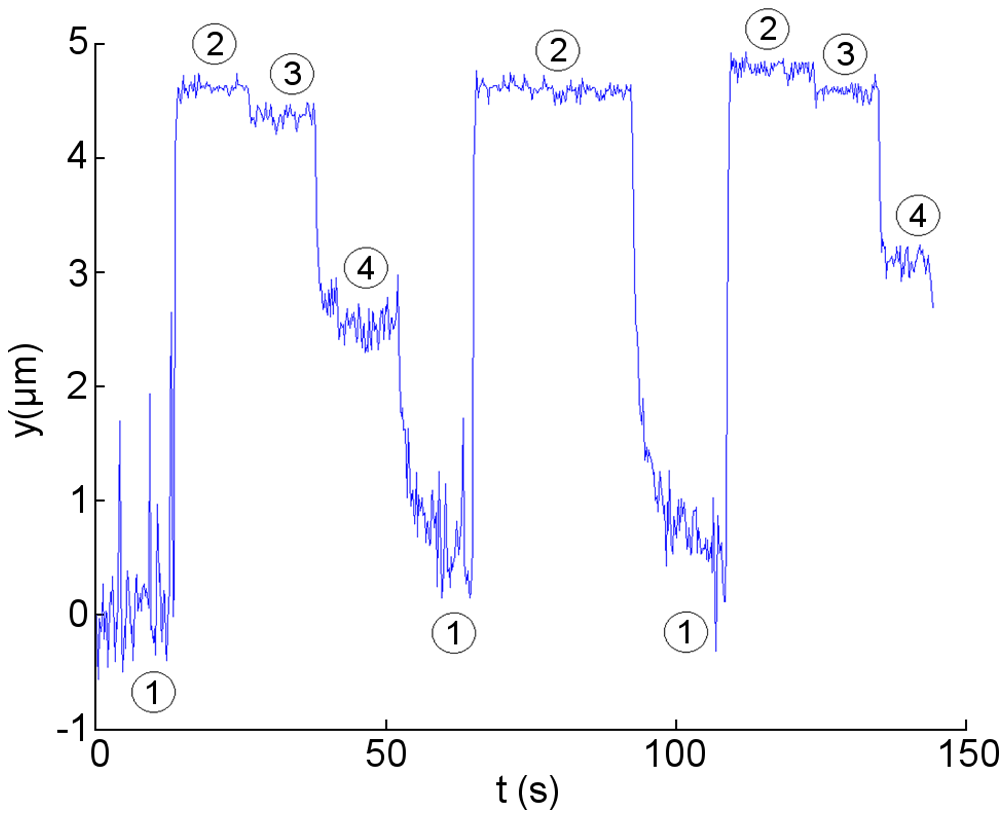
Bacteria move in the direction of and against flow due to pili extension and contraction. Changes in position of an attached bacterium exposed to changes in flow rate. Numbers in circle indicate different shear stresses: (1) → 0.021 pN/µm^2^ (low shear stress), (2) → 0.72 pN/µm^2^ (high shear stress), (3) → 0.41 pN/µm^2^ (medium shear stress), and (4) → 0.062 pN/µm^2^ (medium shear stress).

According to the results obtained in previous AFM experiments, the axial stiffness of pili can be assumed to be *k* = 2 pN/nm [32]. For an applied force *F* = 100 pN, displacement due to a single pilus stretching would be 50 nm according to Hooke’s Law. However, in Figure 1 the attached bacterium moves approximately 4–4.5 µm. This indicates that stretching accounts, at most, for a very small part of bacterial displacements. Taking into account bacterial size, these displacements are not due to bacterial deformation (expected to be ∼500 nm at most) because bacterial displacements up to ∼4.5 µm were observed as in Figure 1, confirming they are due to uncoiling of the quaternary structure of pili.

### Bacteria can Move Significantly in the Direction Opposite to Flow Due to the Effect of Coiling Pili

**Figure 2:**
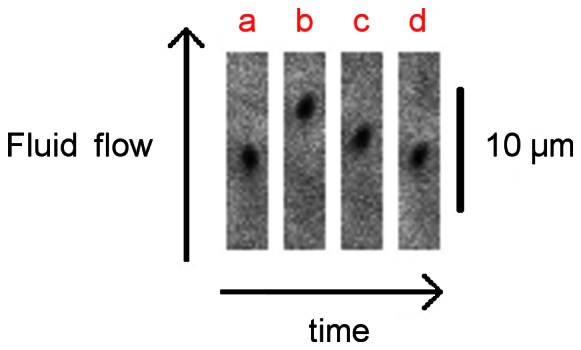
Bacterium moving against flow. Kymograph of microscope images of an attached bacterium under different levels of shear stresses: a – low, b – high, c – low, d – low 11 seconds later.

It has been proposed previously that pili coiling could help bacteria move upstream despite drag forces [15]. In order to verify if this actually occurs under flow, different flow rates are applied producing changes in the level of shear stress (Figure 1): low (1) – high (2) – medium (3) – medium (4) – low (1). When shear stress is increased, bacteria move in the direction of flow due to pili uncoiling as previously explained (Figure 1: t ≈ 15 s, 70 s and 110 s - Figure 2: frames a and b). In the same experiment, when shear stress is decreased, bacterial motion against flow is observed (Figure 1: t ≈ 40 s, 50 s and 90 s - Figure 2: frames b, c and d, supplementary video S1). This result indicates pili can act as a winch when flow rate is reduced, returning to its original coiled state and helping bacteria return approximately to their original position.

### Pili Dynamics

Two dynamic regimes can be discerned in Figure 1 during the retraction process when shear stress is reduced from a medium to a low level (t ≈ 50 s) and from a high to a low level (t ≈ 90 s). The first regime consists of a rapid change in bacterial position just after the flow rate is decreased while the second regime consists of a slower displacement that helps the bacterium nearly reach its initial position (t ≈ 50 to 70 s and t ≈ 90 to 110 s) and is observed at low shear stresses (Figure 1).

#### A first contraction regime is consistent with entropic contraction of the pilin subunits

**Figure 3:**
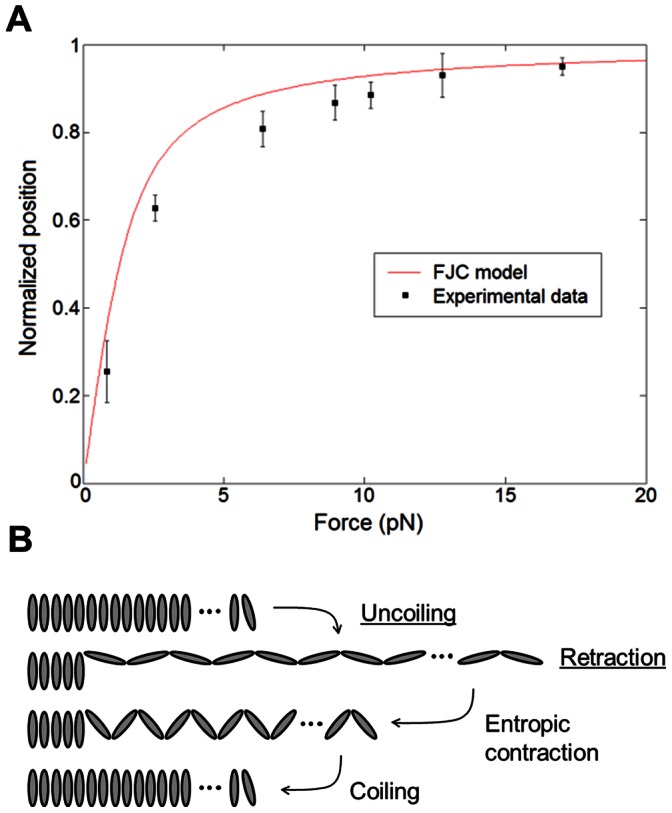
Pili contraction is initially entropic. **A:** Comparison between the normalized Freely Jointed Chain (FJC) model (red curve) and normalized experimental bacterial positions at the end of the first regime for different forces. Error bars represent the standard error of the measurements. **B:** Cartoon showing the changes in the structure of pili for uncoiling and the two regimes in bacterial retraction.

Type I pili consist mainly of a fimbrial rod built from helically coiled FimA (a.k.a. pilin) subunits, each one 5.7 nm long [18]. We model pili as a freely jointed chain (FJC), which is composed of many discrete rigid segments connected by freely rotating joints; the interactions among successive segments are neglected and the chain attains a particular end-to-end length when it is extended by force [33]. Data such as that in Figure 1 from t = 0 to ∼70 s is analyzed. Considering the bacterium moves 4.4 µm when the pilus uncoils (Figure 1: t ≈ 15 s), the FJC model estimates ∼800 segments at this force, assuming each has the length of a FimA protein subunit. In Figure 3A, the red curve indicates the normalized FJC model and the squares represent the normalized experimental bacterial positions averaged for each corresponding. These results strongly suggest that in the first regime, FimA subunits do not coil but rapidly (∼1 s) find a favorable configuration from an entropic point of view. Interestingly, this contraction occurs at intermediate fluid flows (Figure 1), suggesting that the uncoiled state of pili participates in the damping of changes in fluid flow through an entropic spring mechanism.

In the second regime, coiling of the structure is expected since bacteria return close to the original position and increasing the shear stress again, shows the same uncoiling process as before (Figure 1). Figure 3B is a cartoon representing the changes in the structure of pili during the uncoiling and contraction processes.

#### Coiling and recoiling dynamics under flow are different from those observed in single molecule experiments

**Figure 4:**
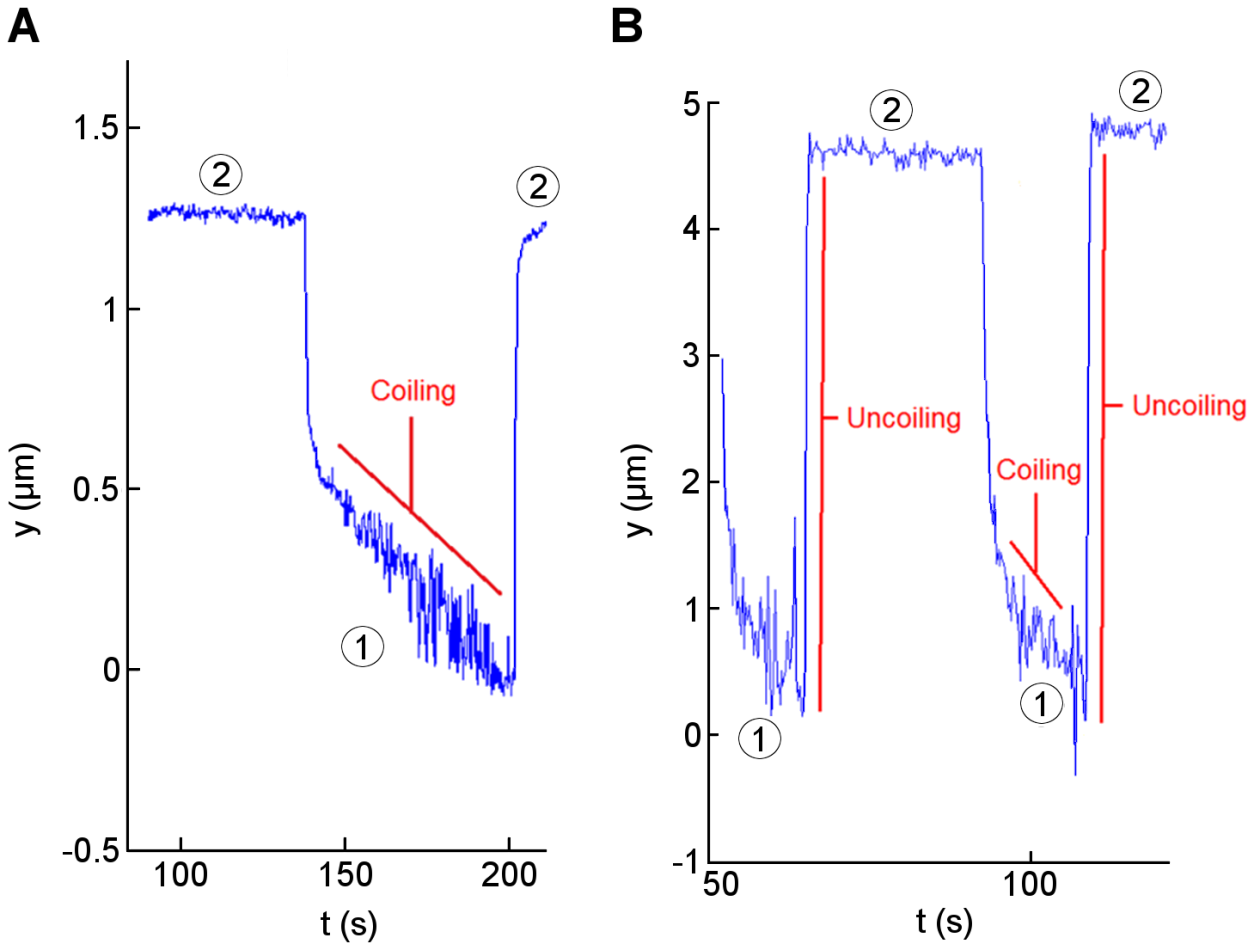
The second contraction regime of pili due to coiling is slow. **A:** Experimental coiling velocity for one bacterium: −9.8 nm/s at 0.66 pN. **B:** Experimental coiling velocity for another bacterium: −41.52 nm/s at 0.66 pN and experimental uncoiling velocities: 7.57 µm/s (t ≈ 70 s) and 7.25 µm/s (t ≈ 110 s) at 23.2 pN. Numbers in circles correspond to the shear stresses defined in Figure 1.

Experimental coiling velocities in the second regime were calculated for different applied forces and compared with a coiling model, derived from AFM measurements [15]. For two different bacteria, the coiling velocities calculated at *F* = 0.66 pN (low shear stress) were estimated at −9.8 nm/s and −41.52 nm/s (Figure 4A and 4B respectively). These values are significantly smaller than the value expected for bacteria adhering via a single pilus, in the order of meters per second according to [15]. Friction forces between bacteria and the surface, bacterial separation, the angle of pili with respect to the applied force or attachment through more than one pilus could cause these slower coiling velocities as described below in the discussion section.

Experimental uncoiling velocities were also calculated and compared with previous models [11], [15] derived from single molecule measurements. From the time-position curves of different bacteria, the uncoiling velocity was calculated to be 8±1 µm/s at *F* = 23.2 pN (high shear stress). According to the previous models, the uncoiling velocity at that force would be three orders of magnitude slower. These results indicate that experimental uncoiling velocities under fluid flow are much faster than the uncoiling velocities predicted by the models.

## Discussion

Our results show that pili coiling and uncoiling happen under flow. Although these results are consistent with previous force spectroscopy experiments done with AFM and OT, the dynamics do not appear to be the same: uncoiling is faster and coiling is slower than in force spectroscopy experiments.

A possible explanation for the slower coiling and faster uncoiling velocities would be an underestimation of the force acting on bacteria. According to the model in [15], the required forces to explain the observed velocities would be 18.7 pN instead of 0.66 pN (the calculated drag force at that shear stress) and 122.4 pN instead of 23.2 pN for coiling and uncoiling, respectively. To calculate the drag force from the shear stress, we initially use the formula from Goldman et al. [34], *F* = 1.7 • 6 π *τ r* ^2^ which describes the force *F* on a stationary sphere of radius *r* which is in contact with the surface in a uniform shear flow (shear stress *τ*). Even though *E. coli* are usually rod-shaped, the growth conditions used in our experiments (as in [32]) generated a bacterial aspect ratio of 1.50±0.05; thus, the errors introduced by the spherical model are not sufficient to explain the discrepancies in the dynamics.

Notably, the value of *r* strongly influences the value obtained for *F* for a given shear stress *τ*. Using a radius of 1.5 µm instead of 0.5 µm increases the force by a factor of 9 for a given shear stress since the value of *F* depends on *r* ^2^ (*F_Gi_* in supplementary Table S1). Both values for the radius are used in the literature. The typical size of *E. coli* is 1 µm wide and up to 3 µm long. The actual size of the bacterium of Figures 1 and 4A is of 2 µm by 1.3 µm. We perform the subsequent calculations using *r* = 0.5 µm and *r* = 1.5 µm as the limiting values, and 1 µm as the average value (supplementary Table S1).

According to Goldman et al. [34], the drag force on an immobilized spherical particle near a plane wall, caused by a uniform shear flow depends, not only on the radius of the sphere *r* but also on the distance between the surface and the underside of the sphere, named *δ*. Consequently, the pre-factor of 1.7 in the initial Goldman formula -for which *δ* is 0- will change depending on both those distances. For a sphere with a radius of 1.5 µm sitting 0.5 µm away from the surface and exposed to a shear stress *τ*, the formula would be *F* = (9/4) • 2.0 • 6 *π τ,* resulting in a pre-factor of 1.17 (*F_Gc_* in supplementary Table S1). Similarly, for a 0.5 µm sphere 0.5 µm away from the surface, the corresponding formula would be *F* = (1/4) • 2.7 • 6 *π τ* and the pre-factor, 1.59. Analysis of the different correction factors shows that both the radius and the distance between the sphere and the surface influence the calculated value of the force acting on the bacterium, with the radius being most critical. However, this adjustment of the formulas to our experimental conditions still does not explain the observed dynamics because the corrections do not result in velocity changes of three orders of magnitude (supplementary Table S2).

Another parameter that could explain the slow retraction velocity is the additional velocity of the bacterium with respect to the chamber during contraction of pili. When a bacterium moves against flow, the velocity with respect to this flow is composed by the velocity of the fluid at a certain distance from the surface of the chamber plus the observed retraction velocity of the bacterium with respect to the chamber. However, this last velocity is in the order of 10’s of nanometers per second (Figure 4), a value three orders of magnitude smaller compared with the fluid velocity at that shear stress, 41.2 µm/s.

**Figure 5:**
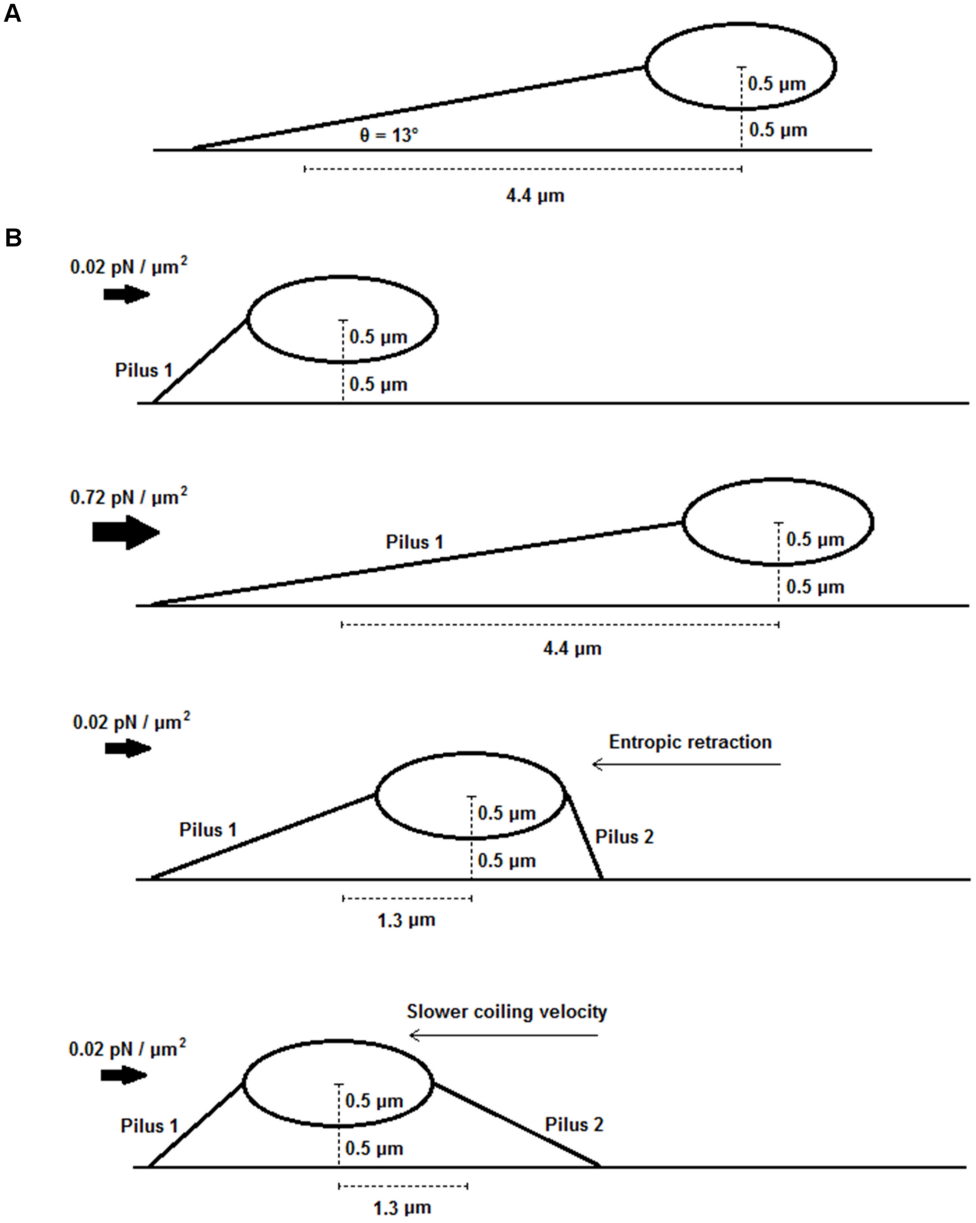
Slow coiling may be due to bacterial separation from surface or a second attached pilus. **A:** Initial angle when shear stress is reduced to a low level (0.02 pN/µm^2^). **B:** Cartoon of a bacterium exposed to an increase and decrease of shear stress, attached by an opposite pilus (pilus 2) which slows the coiling velocity of pilus 1 in the second regime.

We have assumed so far that drag forces act on perfectly horizontal pili. However if a pilus is at an angle, the actual tension on it will be greater due to geometrical factors. Using the data from Figure 1, we calculated an angle of 13° as shown in Figure 5A, corresponding to a correction of the force from 0.26 to 0.27 pN for the lower shear stress. Other cases, presented in supplementary Table S3, show that the changes in the angles of a single pilus do not explain either the difference in dynamics between AFM and flow chamber assays.

**Figure 6:**
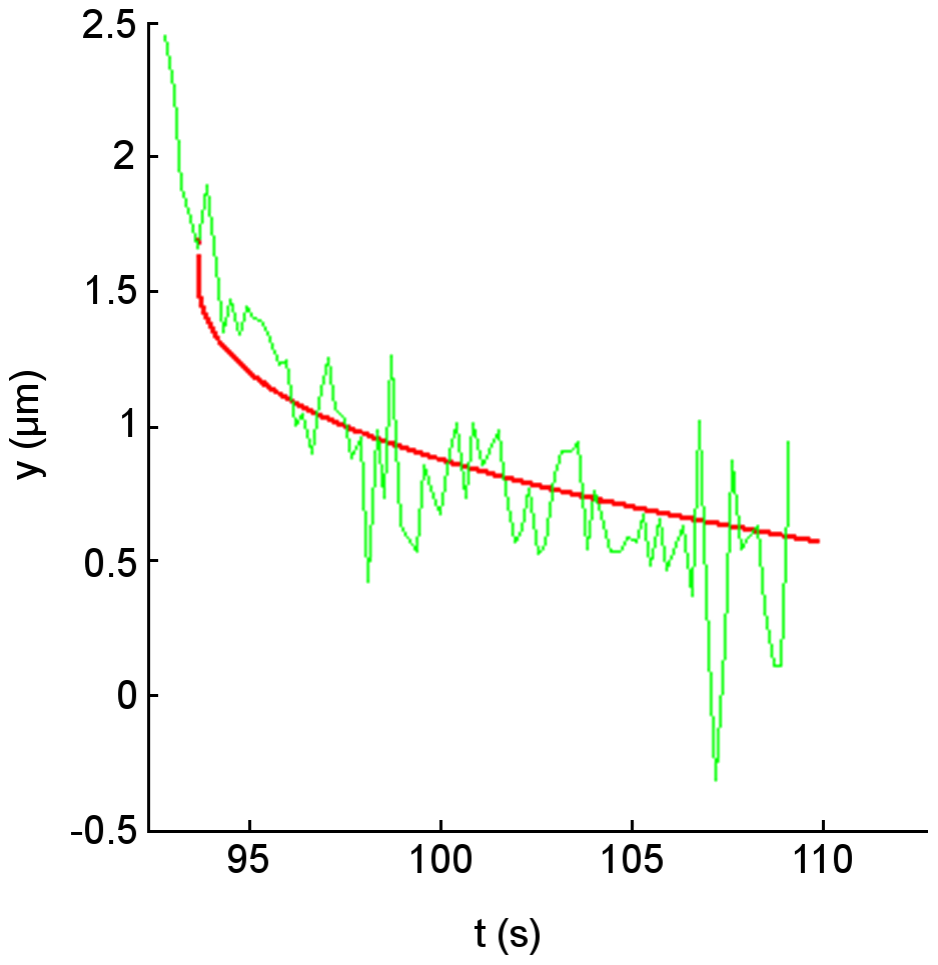
Simulation of the simultaneous coiling and uncoiling of two pili is consistent with experiments. Experimental data from Figure 1 (green curve) compared with the model of a second pilus limiting the coiling process (red curve).

Despite working with a minimal concentration of receptors on the surface of the parallel flow chamber, it may be possible that a pilus at the opposite side of the bacterium limits the coiling process of the first pilus (i.e., in the second regime) resulting in a slower velocity (Figure 5B). We simulated this situation using the coiling and uncoiling parameters from [15] at the corresponding shear stress of the coiling regime to be fitted (Figure 1, t ≈ 100 s). Given that the bacterium moves 1.3 µm during the coiling regime, appropriated values were used: *r* = 1 µm for a bacterium 1 µm away from the surface, 82° between the surface and the opposed ‘braking’ pilus and an initial length of 3 µm for the coiling pilus. Setting these initial conditions, a curve for the position of the bacterium was obtained showing a similar behavior to our experimental results (Figure 6). A chi square goodness-of-fit test indicates that the model represents the experimental data (χ^2^ = 7.37, significance level α = 0.05, four degrees of freedom).

### Biological Significance

Although the results presented here show that bacteria are able to move against flow, it is unlikely that this mechanism alone would allow them to ascend up the urethra since to do so would require a previously formed attachment upstream. Also, uncoiling was not necessary in simulations that reproduce shear stabilized rolling [32]. It has been proposed that the uncoiling of pili is important to resist dislodgement of bacteria from surfaces by working as a shock absorber [15], [30], [35]. This functionality has as well been attributed to the byssal fibers in mussels; then, by comparing the properties of pili with those of byssal fibers it is possible to gain insight into possible mechanisms by which pili resist these fluid forces.

Much like bacteria, mussels use proteinaceous tethers to resist strong fluid forces in order to remain attached to the substratum. The similarity in structure with that of pili is remarkable. Each of these threads, called a byssus, has three different regions: a terminal adhesive region, a stiff distal region and a proximal region that is soft relative to the distal region and more extensible [36]. The terminal region of mussels, which attaches the byssal threads to the substratum, is called the adhesive plaque. It has a counterpart in bacteria, the adhesive tip of pili, which attaches to mannose on the surface of uroepithelial cells. The proximal region of the byssal threads, which is soft and rubbery can be associated with the uncoiled region of pili, which behaves like an entropic spring as shown; the distal region of the byssus can be associated to the coiled section of the pilus due to their relative stiffness with respect to the softer, uncoiled region (see Table 1). The similarity of the two structures in their three-component configuration (adhesive, soft and hard) suggests that all three components play an important role in helping bacteria stay attached to surfaces.

**Table 1:** Mechanical properties of type I pili.

Mechanical properties	Uncoiled region	Coiled region	References
Extensibility (no units)	∼7	0.06 before yield, 7 otherwise	[11], [15], [18]
Toughness (MJ/m^3^)	∼10	∼2	[11]
Strength (MPa)	>45	∼1 (Yield strength)	[15], [44]
Resiliency (%)	∼100% near equilibrium	∼25% to 40% depending on loading rate	[11], [15]
Stiffness (MPa)	≤1 below ∼10pN		This work
	∼10 below ∼30pN		This work
	∼250 between 30 and 80 pN		[15]
	≥200 at 60–150 pN		[11], [15]
		2000	[33]

Further insight about the role of the coiled and uncoiled sections and how they relate to each other can be gained by examining their mechanical properties. We estimate and calculate several mechanical properties that are often used to characterize elastic proteins, and compare them to those of known elastic proteins [37]. Table 1 summarizes the estimates of these mechanical properties for pili; details on the calculations of these properties are explained in the Materials and Methods section.

The extensibility, defined as the ratio between the maximum change in length and the original length when a material is extended, is close to 7 for type I pili. This extensibility is large if we compare it to that of other elastic proteins, and in particular it is more than triple that of the proximal region of the byssus [37]. One proposed function for the extensibility of pili is to scan the surface in order to find additional receptors during the extension process [15]. This exceptional extensibility of pili is consistent with this idea as it facilitates large bacterial displacements, maximizing the chances for attachment. The model introduced in Figure 6 to explain our results is consistent with this function: when bacteria reach their equilibrium position both pili remain partially extended.

Bell and Gosline proposed two important functions for the extension of the distal region of byssal threads: first, the reorientation of threads in the direction of the applied load and second, the distribution of the applied load over several threads [36]. The large extensibility of bacterial pili is consistent with these two mechanisms. In mussels, the reorientation of threads helps reduce the force on individual threads by their alignment with the force vector. The tension on a fiber can be written as *T* = *F*/cos *θ* where *F* is the force that the fiber has to bear and *θ* is the angle between the force vector and the direction of the fiber. When the fiber is aligned with the force vector, the force acting on it is minimized. As pili extend, they tend to align with the direction of force and tension on them is therefore diminished. Since pili do not have a preferential location on the surface of cells, the alignment gained by a large extension can be advantageous to minimize the force on individual pili.

The toughness of a material measures how much energy is required to break it, and in mussel’s byssal threads it is thought to be important in maintaining their attachment to the substrate [37]. The toughness of the uncoiled state of pili is in the order of 10 MJ/m^3^, similar to that of mussels, which are 35 and 45 MJ/m^3^ for the proximal and distal regions, respectively [37]. These values are superior to the toughness of high-tensile steel which is 6 MJ/m^3^ [37], confirming their role as key structural elements.

The strength of a material measures how much force a section of the material is able to withstand before breaking. The strength of the uncoiled state is in the order of 45 MPa, again similar to that of the proximal region of the mussel’s byssus of 35 MPa [37]. It is surprising that both the toughness and the strength of the tethers of bacteria and mussels are similar, considering the two orders of magnitude variation in elastic proteins [37] and the difference in scale between both organisms.

There are similarities between byssal threads and type I pili in terms of dynamics as well. The dynamics of stretching and contraction in the distal region of byssal threads are fast but they do not immediately return to their original length [38], [39]; partial recovery of mechanical properties such as resiliency is achieved after a few seconds, but full recovery can take days [39]. The time scales in bacteria are clearly much faster, but they also present two characteristic time scales: the fast time scale of the contraction of the uncoiled region which is in the order of a second, and the slower time scale of the coiling process, in the order of 10′s of seconds. This result also suggests that the uncoiled region is optimized to buffer rapid changes of smaller forces while the coiled region damps larger changes in force and adjusts the lengths of pili to assure an equilibrated distribution of force between pili.

Uncoiled pili could help maintain the recruitment of pili at shorter time scales than the coiling/uncoiling process. The distribution of load among pili resulting from their large extensibility has been proposed to improve the lifetime of bacterial attachments [35] and recent work supports this idea in the case of the uncoiling process [29]. If the direction or magnitude of flow changes suddenly, the resulting changes in bacterial position or orientation of an attached bacterium should result in different optimal lengths for each pilus in order to ensure load bearing by all or most pili. These magnitude and directional changes are expected for flow in the ureter [40], and in the urethra. It is clear from the contraction dynamics shown here that coiling would take a significant time to adjust the length of pili, whereas entropic contraction can adjust faster and ensure optimal distribution of forces among pili, at least for intermediate forces.

Type I pili show a second uncoiled state in the force-distance curves as an S-shaped tail. This state is most apparent in another type of pili expressed by *E. coli*, P pili [11], [41]. If we assume that uncoiled pili play a role in damping changes in fluid flow, then it makes sense that this entropic-like spring can be tuned for the particular conditions for which they are optimized. In particular, P pili are expressed in bacteria invading the ureter where peristaltic flow is observed [5] while type I pili are expressed in bacteria invading the urethra where sudden flow changes take place occasionally [7]. The energy used to extend the uncoiled section into the second uncoiled state of a pilus represents 70% of the total energy expended in the elongation of P pili, while in type I pili this energy represents 40–50% supporting the idea that the *uncoiled* section of both types of pili plays a significant role in buffering changes in force. In particular, for P pili it makes sense that if the uncoiled region is important in damping intermediate changes in fluid flow to which they are continually exposed, then they require a majority of the extension energy. Furthermore, diversity in the mechanical properties of pili that colonize diverse flow environments is also observed in mussels. The mechanical properties of byssal filaments can change between different mussel species that colonize areas with different exposures to surf [42] and within single species due to seasonal changes in wave heights [42], [43].

For both bacteria and mussels, dissipation of energy of fluid flow via their tethers has been proposed to help maintain attachment during sudden changes in flow. For bacteria, energy dissipation during uncoiling has been shown to increase the lifetime of adhesion of bacteria to surfaces [30]. One way to quantify the energy dissipated in an uncoil/coil cycle is to measure resiliency, which is the ratio of the energy a fiber returns during contraction as a fraction of the energy required for extension. It is possible to calculate the resiliency of pili from previous studies near equilibrium performed with OT [11] and further away from equilibrium performed with AFM [15]. In the case of type I pili, this is in the order of 25% under dynamic AFM conditions and closer to 40% in near-equilibrium conditions. These values are similar to that of the distal region in mussel’s byssal threads, at 28%, and clearly support the idea that the dissipation of energy is a relevant property in pili.

The resiliency in byssal threads is also dependent on the loading rates and diminishes further from equilibrium [39]. In both mussels and bacteria, as the loading rate increases, the resiliency of the tethers decreases and more energy is dissipated, thereby diminishing the energy available to break the terminal adhesion. A decrease in resiliency can be deduced from the data in [15] for bacteria: the uncoiling force increases with increasing loading rate and the coiling force decreases at increased negative loading rates. Resiliency is load rate dependent, and decreases with increasing loading rate, explaining the difference in resiliency between the OT and AFM measurements. This decrease in resiliency with increasing dynamics may be inherent to systems where bonds are broken sequentially during extension. Experiments, simulations and analytic expressions based on Kramer’s theory have established that the breaking force of a single bond increases with loading rate. If bonds break sequentially we should have the same breaking event at the given loading rate repeated over the sequential bonds. In the case of negative loading rates it makes sense that the converse is the case, and the results in [15] support the idea. A consequence of this is that for the uncoiled region, which appears to have a resiliency close to 100% near equilibrium, we should expect a decrease in resiliency as loading rates (cycling frequency) increase. Therefore, in highly dynamic environments uncoiled pili should dissipate more energy, and improve their damping properties, complementing the damping properties of uncoiling.

One important difference between pili and byssal threads is that the strength before yield of coiled pili is particularly small relative to the uncoiled strength before breaking. The force at which type I pili start uncoiling significantly (yielding), around 50 pN near equilibrium [11], is low compared to the 140 to 180 pN at which pili or their terminal bonds break [44]. In mussels, the distal region starts yielding at forces slightly lower than the breaking forces of the proximal region, most likely to prevent it from breaking. When pili extend, two plateaus can be discerned on the force-extension curve. The first corresponds to the uncoiling of pili, and is flat. The second plateau is not as flat and has been attributed to the transition from the first uncoiled state to a second uncoiled state, forming an S-shape in the force curve. A barrier, presumably due to head to tail interactions between the uncoiled subunits, separates the two states [41]. This second plateau can be observed at 68pN near equilibrium, yielding at a force nearer the breaking force of the terminal bond than the uncoiling plateau. The yield of the distal region in mussels is slightly below the breaking force of the proximal region and the adhesive region, suggesting that its function is to yield before these other regions break. Here we suggest that the second plateau is likely to perform this protective role in pili, at least for partially or fully uncoiled pili.

Coiling and uncoiling dynamics are intimately linked to each other by the equilibrium force which is the force at which coiling and uncoiling balance each other (see equations in [15] for example). A larger uncoiling force would result in a higher equilibrium force. This higher equilibrium force would promote the breakage of the terminal adhesin because pili are able to exert a coiling force close to the equilibrium force, which would be larger than the optimal catch bond force. When a bacterium is attached via multiple pili, an equilibrium position should be reached when all the attached pili arrive close to the equilibrium force. Indeed, this was observed in the simulation of Figure 6: the force when the bacterium came to a halt was close to 20 pN for both pili, which is lower than that at which the lifetime of the FimH complex is maximal, near 30 pN [25], [26]. However, it is close to the maximum lifetime of the strongly bound state, which is lower than that of the complex and closer to 20 pN as shown in [25]. Consequently, this equilibrium force could maximize the lifetime of strongly bound pili and, by extension, the attachment lifetime of the bacterium.

The value of the equilibrium force is consistent with the functionality of the uncoiled state of pili. An intermediate coiling force has the consequence that a significant portion of pili remain uncoiled when there are multiple attachments, ensuring that the uncoiled section performs its functions. Another possible consequence of an intermediate coiling force is that adhered pili are always recruited into tension ensuring that a maximum number of pili are active in subsequent changes in flow. Finally, by looking at the relationship between coiling and equilibrium forces, we note that if the uncoiling force were larger, it would be possible to restore the equilibrium force to its original value at the expense of slower coiling velocities. This could be interesting for bacteria in cases of repeated surges in flow. Bacteria are probably required to find a balance between the uncoiling force, the velocity of coiling and the equilibrium force of pili to maintain maximal attachment.

### Conclusions

We present evidence of coiling and uncoiling of pili under flow by analyzing the displacement of surface-bound bacteria exposed to changing levels of fluid flow. Bacteria are able to move against flow due to contractions in the length of pili, which display two different dynamic regimes: A first contraction regime is consistent with an entropic spring, suggesting FimA subunits are relaxing but not coiling back to their original state. A second contraction regime is slower than that expected from AFM measurements but consistent with a model of a coiling pilus limited in its coiling velocity by a second extending pilus. Our experimental data indicate that coiling velocities –measured in the second contraction regime observed– are slower while uncoiling velocities are faster than those derived in AFM and OT experiments, which were taken in conditions closer to equilibrium.

The two contraction regimes observed suggest that the coiled and the uncoiled regions have different functions much like byssal threads in mussels with which they share structural similarities. Surprisingly, the toughness and strength are large and similar between pili and byssal threads, confirming their importance as attachment structures. The extensibility of pili is exceptional when compared to that of many biological fibers and is consistent with two functions previously proposed in mussels: the alignment of threads in the direction of fluid flow that helps reduce force on individual threads and the distribution of force among threads. These functions of pili would complement the function of dissipating energy during uncoiling in order to protect the terminal bond, a function that is likely performed by the second uncoiled state of pili. Moreover, we argue that the functions so far attributed to uncoiling, namely damping, recruitment into tension and alignment are complemented by the uncoiled state at intermediate forces and high dynamic ranges. The resulting natural composite tether material is able to address a wider range of functions than a simple tether.

Overall, our results and analysis add to the understanding of how coiled and uncoiled pili may be interacting to optimize the forces on individual pili and maximize bacterial adhesion under flow. Simulations that explicitly include the second uncoiled state and further experiments to study the dynamics of pili would help support the functions proposed here.

## Materials and Methods

### Bacteria and Growth Conditions

Uropathogenic *E. coli* bacteria were maintained on Super Broth (SB) at 4°C until experimentation when they were grown in SB for 16 hours prior to harvesting. After 16 hours, bacteria are in their stationary growth phase where they show greater adhesion to human uroepithelial cells *in vitro* [45]. Bacteria were harvested by centrifugation (5 min at 4300×*g*), washed three times with phosphate buffered saline (PBS) solution, and resuspended in PBS with 0.1% bovine serum albumin (BSA).

### Flow Experiments

35 mm plastic petri dishes were functionalized with 50 µL of Ribonuclease B (RB) from bovine pancreas obtained from Sigma (R7884) (St. Louis, Missouri, United States) at the desired concentration (see below) in a 0.02 M bicarbonate buffer and incubated for 1 h at 37°C. Petri dishes were then rinsed three times in PBS with 0.1% BSA (PBS-BSA) in order to reduce nonspecific binding and incubated at 37°C for 30 min with 1 mL of PBS-BSA. RB is a model tri-mannose (3M) receptor that binds strongly to FimH, the adhesin found in *E. coli* type I pili [46].

Shear stresses were generated using a parallel plate flow chamber and gasket (GlycoTech®) sealed onto a 35 mm Corning plastic petri dish coated with RB at concentrations between 0.01 and 0.1 µg/mL. *E. coli* bacteria dispersed in PBA-BSA were injected with the desired flow rate using a WPI SP120p syringe pump (World Precision Instruments Inc. – Sarasota, Florida, United States). For most of our experiments a concentration of 0.02 µg/mL RB was used, where a minimal number of bacteria were attached but where some attachment was still present.

Bacteria were observed under an inverted optical microscope (Zeiss Axiovert 40 CFL). Initial experiments of bacterial displacement were recorded with a Moticam 1000 camera via Motic Images Plus video acquisition software (Motic Group – Hong Kong, China) at 17 fps and later experiments with a CoolSnap EZ camera (Photometrics – Tucson, Arizona, United States) via Micromanager video acquisition software (µManager – San Francisco, California, United States) at 17 or 5 fps. The field of view was calibrated for each camera using a calibration slide from Motic in order to convert pixels to nanometers.

### Image Analysis

Two different methods for image analysis were developed which yielded similar results, but had improved tracking (did not lose bacteria) for each of the two cameras. Freely jointed chain extension and displacement measurements were performed with the first method, while the other figures were obtained using the second method. The methods were validated with respect to each other by running them on some of the same videos.

#### Method 1

Briefly, bacteria were tracked using an in-house algorithm based on the 2D cross correlation algorithm. This algorithm locates a bacterium, which is identified in the first frame of the video, in subsequent frames to determine its instantaneous displacement. Details follow.

Given each frame in a matrix *A* with dimensions (*M,N*) and the template of the bacterium from the first frame in a smaller matrix *T* with dimensions (*m*1,*n*1), the algorithm computes the normalized two-dimensional discrete cross-correlation coefficient matrix. This matrix has its maximum value in the location of matrix *A* which more closely resembles the template *T*.

The cross-correlation matrix is calculated using the formula [47]:
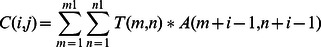
where 1< *i*<*M* − *m*1 and 1< *j*<*N* − *n*1. Finally the coefficient matrix is normalized, 
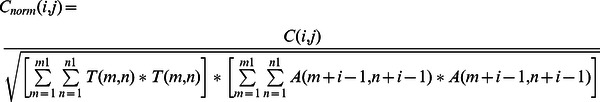



The location of the maximum value in matrix *C_norm_* corresponds to the location of the bacterium in the current frame.

#### Method 2

Black and white grayscale images from the movies are individually analyzed. First, the bacterium is segmented; a region of interest is chosen to enclose the bacterium. A threshold is applied to the image identifying the darkest pixel values that correspond to the bacterium. Morphological operations are applied to the binarized image in order to clean up the image. The bacterial region is Opened and Closed [48], eliminating isolated points in the threshold image not corresponding to bacteria. Once connected regions over a pixel in size are obtained, the shrink operation is applied until the bacterium is shrunk to a point corresponding to its centroid. The centroid is used as seed to do a region growing and only the connected regions with an area greater than the value initially found are considered as bacteria. Coordinates of the center of mass of each connected region or bacterium are calculated providing the location of the bacterium in each frame.

### Calculation of Forces Acting on Bacteria

The relation between force *F* (pN) and shear stress *τ* (pN/µm^2^) is given by the formula (*F* = 1.7 • 6 *π τ r* ^2^) according to Goldman et al. [34] taking a radius *r* of 1 µm for bacteria. This formula was used for the forces indicated in Figure 4. The relation between flow rate *Q* and shear stress *τ* is given by *Q* = *τ a* ^2^ *b*/6*µ* where *a* is the channel height (250 µm), *b* is the channel width (0.25 cm) and *µ* is the dynamic viscosity. We took *µ* to be the viscosity of water at 20°C (0.01 Poise).

### Freely Jointed Chain Model

The chain is composed of *n* straight segments; each segment has a length *b* and can point to any direction with equal probability. For a force *F* applied to a chain, the distance the chain extends is given by <*x*> = *n b* L(*Fb*/*kT*) where *k* is the Boltzmann constant, *T* is the temperature and L is the Langevin function defined as L(*z*) = coth(*z*) –1/*z* [33]. In a bacterial pilus, *n* represents the number of subunits (FimA) which makes up the pilus and *b* is the length of one subunit (5.7 nm [18]). In Figure 3A, in order to average data from bacterial pili with different number of subunits, the position of the bacterium -representing the extension of the pilus- was normalized dividing <*x*> by *nb*.

### Calculation of Mechanical Properties of Uncoiled and Coiled Pili

#### Extensibility

For whole pili, the maximum extensibility can be approximated by taking data from the electron microscopy structure [18]: a FimA monomer contributes 0.7 nm when coiled but it is 5.7 nm long, therefore its extensibility is (5.7–0.7)/0.7 ≈ 7. By analyzing AFM and OT data we calculate that pili only extend 0.06 before uncoiling [11], [15] so most of the extensibility is due to the uncoiled state of pili.

#### Strength

Strength is estimated for the uncoiled region by dividing the estimated average breaking forces observed from constant velocity pulls in [44] (between 140 and 180 pN, we used 150 pN for our calculations) by the expected cross sectional area of a FimA monomer (the diameter was taken as ∼2 nm and the monomer was assumed to have a circular cross section). The rupture force used here corresponds to the breaking of the FimH bond with mannose. The strength of pili, given by the strength of the donor-strand complementation of FimA monomers, is expected to be larger because rupture is due to receptor-ligand dissociation, as shown previously [15]. How much larger is hard to quantify since on the one hand, there should be no reason for pili to be mechanically over-designed with respect to FimH, and on the other hand, the bonds that hold pili together have been shown to have exceptional kinetic stability with respect to unfolding and dissociation [19]. Either way, since the maximum force that pili can withstand when attached to a surface is limited by the terminal adhesive, it is reasonable to use that force, even if pili themselves are stronger.

For the coiled region we consider the yield strength, which is the strength at which the material stops extending linearly, which in this context is the strength at which uncoiling is initiated. The yield strength of the coiled region is small at around 1 MPa, when we consider the force at which uncoiling begins (∼30 pN [15]) and the area of a circular cross section using the diameter of a coiled pili (7 nm).

#### Stiffness

For the uncoiled region, first its spring constant is calculated from the force-extension curves when pili are modeled as freely jointed chains (before normalization). Since the curve is nonlinear, we estimate three spring constants for different forces: for low forces (below ∼10 pN), medium forces (below ∼30 pN) and high forces (between 60 and 150 pN). Then, the Young modulus was calculated as *E* = *k L*/*A* (Table 1) where *k* is the spring constant, *L* is the extension of the chain for the particular force and *A* is the cross sectional area of a FimA monomer, which was assumed to have a ∼2 nm diameter. Alternatively, the Young modulus is also calculated for higher forces from the nonlinear region of the force-extension curves from single pili experiments [11], [15]: an uncoil/coil cycle is analyzed (figure 2B in [15]), which provides the spring constant of the partially uncoiled pilus and then, the Young modulus of the uncoiled region. For coiled pili, no appropriate measurements have been made since the spring constant of the tip is low compared to that of the coiled region; then, like Whitfield et al. [32] we estimate that the stiffness is comparable to a stiff protein [33].

#### Toughness

For the uncoiled region, toughness is estimated by first approximating the energy required to fully extend *uncoiled* pili, which corresponds to the final area under the force-extension curve (the nonlinear S-like region) as well as part of constant force region corresponding to the area under a freely jointed chain extension curve, which we approximated by extrapolating down the ‘S’ region of the curve (supplementary Figure S1A). The resulting energy is divided by the volume of the fully extended pilus (cross sectional area of a FimA monomer (∼3,14 nm^2^) times the length of the pilus when force reaches 150 pN).

A measurement of toughness for the coiled region should include the energy until breakage. However, since there is a transition into the uncoiled state, which is being characterized separately, it is not clear how to calculate it; we therefore include the energy of the uncoiled state (supplementary Figure S1B). The total energy is divided by the volume of the coiled pilus (cross sectional area of a coiled pilus (∼38 nm^2^) times the length of the pilus when completely coiled). Figures from [11] are used to estimate the energy in both regions.

#### Resiliency

Resiliency is estimated based on the ratio of the extension and contraction forces in force-extension curves close to equilibrium and in dynamic conditions. OT measurements near equilibrium, show an uncoil/coil cycle with no hysteresis in the uncoiled region (region III) and a lower force during coiling with respect to uncoiling which results in a resiliency of ∼40% (figure 2B from [11]). In the case of AFM measurements, further away from equilibrium, the resulting resiliency is of ∼25% (figure 2B in [15]).

## Supporting Information

Figure S1 **Toughness calculation for type I pili.** A schematic illustration of typical force-elongation curves where the red region indicates the energy used to calculate toughness as energy/volume. **A:** For the uncoiled region, an extrapolation of the uncoiled state is made down to 0 pN. **B:** For the coiled region, the energy from all the elongation process until breakage is used.
(TIF)

Table S1 **Force correction factors for fluid drag near a surface.**
(DOCX)

Table S2 **Calculated forces taking into account the distance between the sphere and the surface.**
(DOCX)

Table S3 **Changes in the force acting on a pilus are not significantly different between a horizontal pilus and one at an angle.**
(DOCX)

Text S1 **Control: Bacteria move in the direction of flow while attached to the surface via pili.**
(DOCX)

Video S1 **Movie showing displacement of an attached bacterium due to changing fluid flows.**
(MP4)
