# Supplementary figures and images for: Observation of Bacterial Type I Pili Extension and Contraction under Fluid Flow
Source: PLoS One. 2013 Jun 14;8(6):e65563. doi: 10.1371/journal.pone.0065563 (PMC3683016; doi:10.1371/journal.pone.0065563)

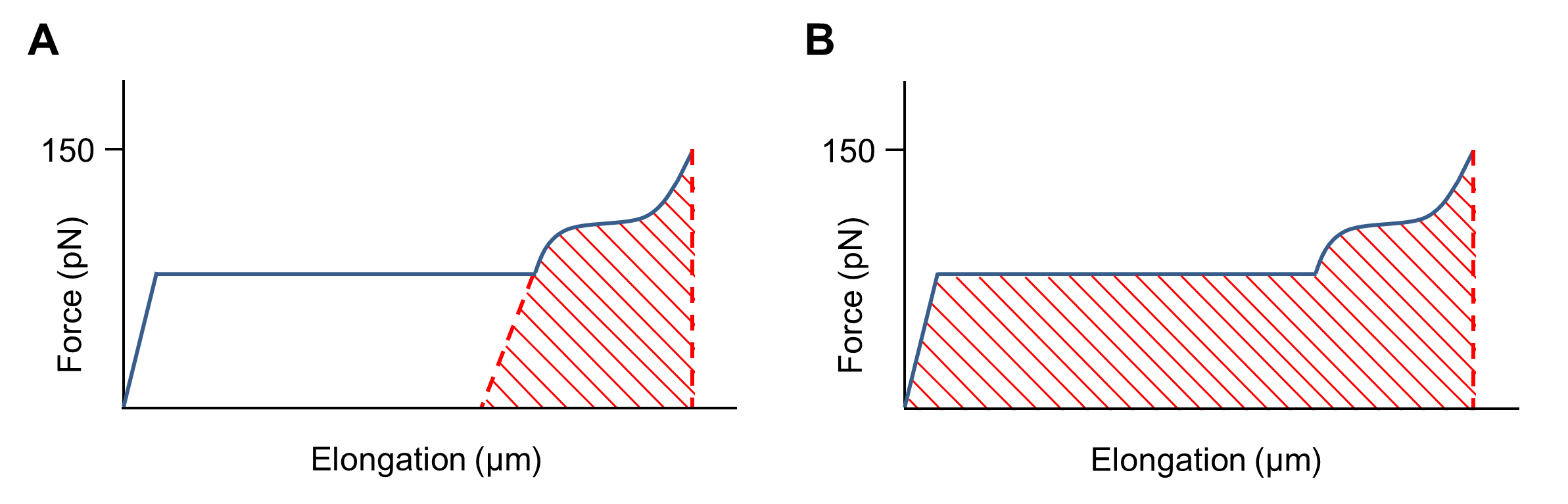

Supplement: Figure S1 — Toughness calculation for type I pili. A schematic illustration of typical force-elongation curves where the red region indicates the energy used to calculate toughness as energy/volume. A: For the uncoiled region, an extrapolation of the uncoiled state is made down to 0 pN. B: For the coiled region, the energy from all the elongation process until breakage is used. (TIF) [file pone.0065563.s001.tif]
