# Supplementary material for: Observation of Bacterial Type I Pili Extension and Contraction under Fluid Flow
Source: PLoS One. 2013 Jun 14;8(6):e65563. doi: 10.1371/journal.pone.0065563 (PMC3683016; doi:10.1371/journal.pone.0065563)
Supplement: Table S1 — Force correction factors for fluid drag near a surface. (DOCX) [file pone.0065563.s002.docx]

**Table S1. Force correction factors for fluid drag near a surface.**

|  | ***r* = 0.5 μm** | ***r* = 1 μm** | ***r* = 1.5 μm** |
| --- | --- | --- | --- |
| *δ* = 0 μm* | $F_{Gi}=\frac{1}{4}1.7\cdot6\pi\tau$ | $F_{Gi}=1.7\cdot6\pi\tau$ | $F_{Gi}=\frac{9}{4}1.7\cdot6\pi\tau$ |
| *δ* = 0.5 μm* | $F_{Gc}=\frac{1}{4}2.7\cdot6\pi\tau$ | $F_{Gc}=2.2\cdot6\pi\tau$ | $F_{Gc}=\frac{9}{4}2.0\cdot6\pi\tau$ |

9 in a correction factor of 1.5istancia superficie - bactria**δ*: surface-bacterium distance

Correction factors for Stokes drag forces near a surface change depending on the radius of the sphere *r* and on the surface-bacterium distance *δ*, for a given shear stress τ. Formulas are given for three typical values of *E. coli* radius: *F_Gi_* corresponds to the force acting on a sphere in contact with the surface and *F_Gc_* corresponds to the force acting on a sphere 0.5 µm away from the surface.
