# Supplementary material for: Observation of Bacterial Type I Pili Extension and Contraction under Fluid Flow
Source: PLoS One. 2013 Jun 14;8(6):e65563. doi: 10.1371/journal.pone.0065563 (PMC3683016; doi:10.1371/journal.pone.0065563)
Supplement: Table S2 — Calculated forces taking into account the distance between the sphere and the surface. (DOCX) [file pone.0065563.s003.docx]

**Table S2. Calculated forces taking into account the distance between the sphere and the surface.**

|  | ***r* = 0.5 μm** | ***r* = 1 μm** | ***r* = 1.5 μm** |
| --- | --- | --- | --- |
| *τ* = 0.021 pN/µm^2^ | *F_Gi_* = 0.17 pN | *F_Gi_* = 0.66 pN | *F_Gi_* = 1.5 pN |
|  | *F_Gc_* = 0.26 pN | *F_Gc_* = 0.85 pN | *F_Gc_* = 1.8 pN |
| *τ* = 0.72 pN/µm^2^ | *F_Gi_* = 5.8 pN | *F_Gi_* = 23 pN | *F_Gi_* = 52 pN |
|  | *F_Gc_* = 9.1 pN | *F_Gc_* = 30 pN | *F_Gc_* = 62 pN |

Force is calculated for *r* = 0.5, 1 and 1.5 μm for two shear stress values, using the two formulae given in Table S1. Taking into account a distance of 0.5 µm between the sphere and the surface, the force acting on the sphere is greater (*F_Gc_*) but still of the same order of magnitude, and does not explain the differences in the dynamics of pili.

9 in a correction factor of 1.5istancia superficie - bactria
