# Supplementary material for: Observation of Bacterial Type I Pili Extension and Contraction under Fluid Flow
Source: PLoS One. 2013 Jun 14;8(6):e65563. doi: 10.1371/journal.pone.0065563 (PMC3683016; doi:10.1371/journal.pone.0065563)
Supplement: Table S3 — Changes in the force acting on a pilus are not significantly different between a horizontal pilus and one at an angle. (DOCX) [file pone.0065563.s004.docx]

**Table S3. Changes in the force acting on a pilus are not significantly different between a horizontal pilus and one at an angle.**

|  | ***r* = 0.5 µm, *θ* = 13°** | ***r* = 1 µm, *θ* = 19°** | ***r* = 1.5 µm, *θ* = 24°** |
| --- | --- | --- | --- |
| $F_{pilus}=\frac{F_{Gc}}{\cos\theta}$ | *F_Gc_* = 0.26 pN | *F_Gc_* = 0.85 pN | *F_Gc_* = 1.8 pN |
|  | *F_pilus_* = 0.27 pN | *F_pilus_* = 0.90 pN | *F_pilus_* = 2.0 pN |

The force on a pilus *F_pilus_* is calculated at *τ* = 0.02 pN/μm^2^ using the formula for *F_Gc_* given in supplementary Table S1 and taking into account the angle between the pilus and the surface for three values of *r*. The force increases but remains in the same order of magnitude, and does not explain either the differences in the dynamics of pili.
