# Supplementary material for: Observation of Bacterial Type I Pili Extension and Contraction under Fluid Flow
Source: PLoS One. 2013 Jun 14;8(6):e65563. doi: 10.1371/journal.pone.0065563 (PMC3683016; doi:10.1371/journal.pone.0065563)
Supplement: Text S1 — Control: Bacteria move in the direction of flow while attached to the surface via pili. (DOCX) [file pone.0065563.s005.docx]

**TEXT S1. Control: Bacteria move in the direction of flow while attached to the surface via pili**

Since a parallel flow chamber is used to recreate the physiological conditions, it is important to discard the possibility that bacterial displacements are caused by changes in pressure inside the chamber when flow rate is changed. These pressure changes could generate a deformation in the chamber surface showing an apparent bacterial displacement under the microscope.

A bacterium attached to the surface was found near a dust particle (Figure S1A) which moved when the stage of the microscope moved confirming that it was on the chamber surface and not somewhere else in the optical path. Three changes in flow rate were applied, corresponding to the following changes in shear stress: 0.021 pN/µm^2^ – 3.1 pN/µm^2^ – 0.021 pN/µm^2^ – 3.1 pN/µm^2^. Near t ≈ 50 s in Figure S1B, the dust particle (red curve) presented no significant displacement when flow was changed as compared with the observed bacterial displacement (blue curve) which was of approximately 1.2 µm (first red arrow). The same result is obtained at t ≈ 200 s (second red arrow). These results confirm that bacterial displacements are not caused by changes in pressure inside the chamber.


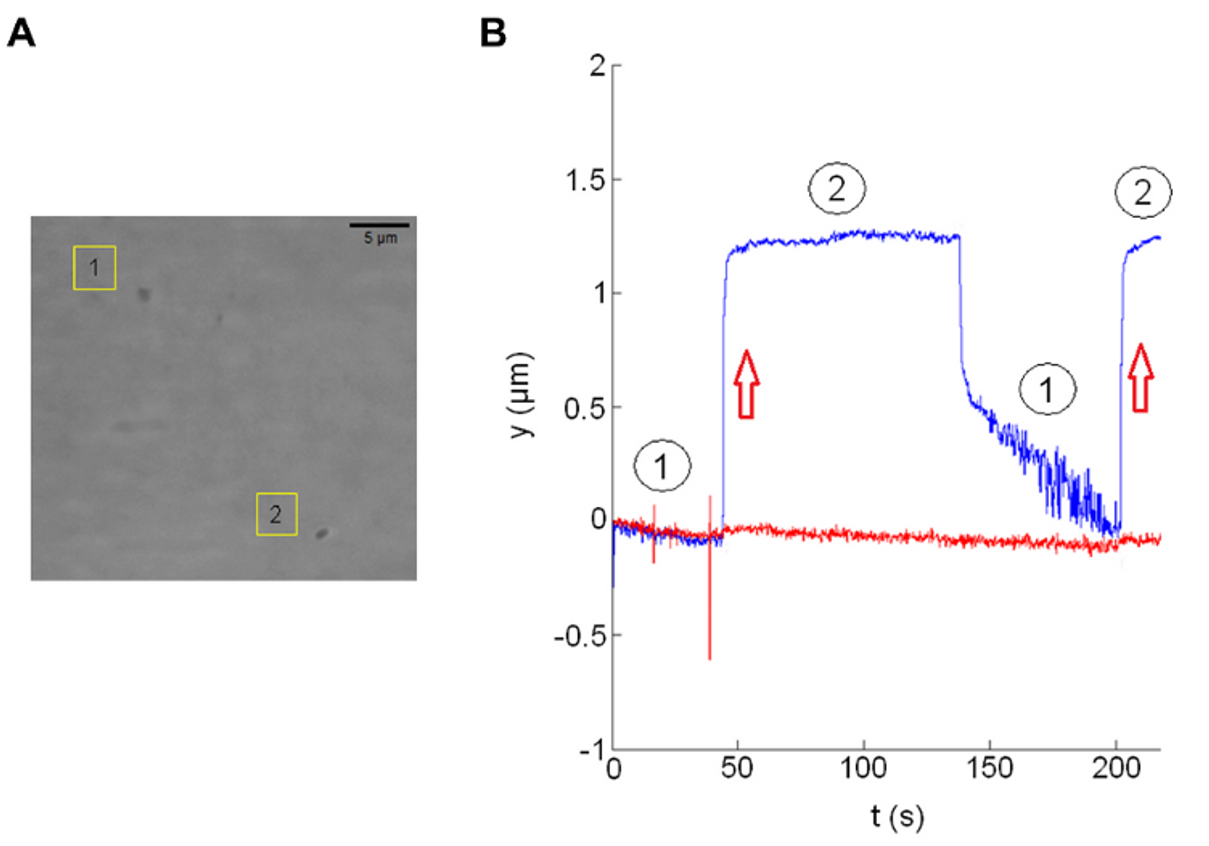


**Figure S1. A:** Microscope image showing the negative control (1) and an attached bacterium (2). **B:** Position *y* (µm) of negative control (red curve) and the attached bacterium (blue curve). Red arrows indicate pili uncoiling. Numbers in circles correspond to the shear stresses defined in Figure 1 in the main text.
